# A Case of Parkinson's Disease with No Lewy Body Pathology due to a Homozygous Exon Deletion in* Parkin*

**DOI:** 10.1155/2018/6838965

**Published:** 2018-06-28

**Authors:** Krisztina Kunszt Johansen, Sverre Helge Torp, Matthew J. Farrer, Emil K. Gustavsson, Jan O. Aasly

**Affiliations:** ^1^Department of Neurology, St. Olavs University Hospital, Trondheim, Norway; ^2^Department of Pathology, St. Olavs University Hospital, Trondheim, Norway; ^3^Department of Neuroscience, Norwegian University of Science and Technology, Trondheim, Norway; ^4^Department of Medical Genetics, University of British Columbia, Vancouver, BC, Canada

## Abstract

Parkinson's disease (PD) is a clinical diagnosis based on the presence of cardinal motor signs, good response to levodopa, and no other explanations of the syndrome. Earlier diagnostic criteria required autopsy for a definite diagnosis based on neuronal loss in the substantia nigra pars compacta (SNpc) and the presence of Lewy bodies and neurites. Here, we present a patient who developed parkinsonism around the age of 20, with an excellent response to levodopa who, at age 65, received bilateral STN deep brain stimulation (DBS). The patient died at age 79. The autopsy showed severe neuronal loss in the SN without any Lewy bodies in the brainstem or in the hemispheres. Genetic screening revealed a homozygous deletion of exon 3-4 in the* Parkin* gene. In this case report we discuss earlier described pathological findings in* Parkin* cases without Lewy body pathology, the current diagnostic criteria for PD, and their clinical relevance.

## 1. Introduction

Parkinson's disease (PD) is the most common movement disorder with a heterogeneous clinical presentation. During the last decades different diagnostic criteria have been applied but in most of them cardinal motor signs with good levodopa response and absence of other explanations of the symptoms were central. The gold standard of diagnosis used to be based on autopsy findings with neuronal loss in substantia nigra pars compacta (SNpc) and the presence of Lewy bodies (LB) and neurites [1, 2]. MDS recently published a renewed clinical diagnostic criterion for PD focused on the clinical signs and no autopsy findings for a more systematical use in clinical practice [3].

Biallelic mutations in* Parkin* (PARK2), including insertions and deletions, are the most common autosomal recessive form of parkinsonism.* Parkin*-associated PD has been frequently described among patients with early onset PD (EOPD) [4, 5] and most* Parkin* cases have a younger age at symptom onset and a slower disease progression with a more benign disease course compared to idiopathic PD. Low dose of levodopa often induces motor complications with dyskinesias. Dystonia in lower limbs and hyperreflexia is not uncommon whereas autonomic dysfunctions and dementia are rare [6]. Lohmann et al. compared patients with EOPD with and without* Parkin* mutations based on neurological, neuropsychological, and psychiatric profiles and could not distinguish the two groups on the individual basis [7]. The clinical spectrum of* Parkin*-associated PD has been widened by different phenotypes including cervical dystonia, autonomic dysfunction, and peripheral neuropathy [8]. Increasing number of studies has reported autopsy findings in* Parkin* mutations carriers, in most cases with isolated neuronal loss in SNpc and no diffuse LB pathology [9–18]. A few studies have reported the presence of LBs in* Parkin* patients, mainly in compound heterozygous [10, 19–21] and in two cases with homozygous mutation carriers [22, 23]. Except for two cases, all LB positive cases had a symptom onset later than age of 40; thus it has been doubted whether it could have been coexisting incidental pathology. A case had atypical basophilic inclusions in PPN and not typical LBs [10, 23].

In this report we present EOPD carrying homozygous* Parkin* deletion of exons 3-4 with no LB pathology at autopsy.

## 2. Case Report

A young man in his early twenties with no prior history of medical treatment started experiencing stiffness in his left leg during physical activity when he did his mandatory military service. A few years later, after a short stay in hospital, he was diagnosed with a functional movement disorder. He had an older sister affected by PD with disease onset in her late forties. Between age of 30 and 40 he was seen by several neurologists as he experienced worsening of asymmetric stiffness, pain, and sensory symptoms in his lower extremities. He was finally diagnosed with PD around age 40. He responded well to levodopa treatment and after many years on levodopa he developed increasing dyskinesias. He managed to stay in his academic position up in his midsixties and underwent STN DBS at age 65 because of medically intractable dyskinesias. He lived at home with support of health care assistance until his death at age 79. He did not show any sign of dementia.

Multiplex ligation-dependent probe amplification (MLPA) analysis revealed a homozygous deletion of exons 3-4 in the* Parkin* gene [24].

## 3. Autopsy Findings

The macroscopic exam of the brain did not show any sign of cerebral atrophy, no dilated ventricle system and no sure sign of hypopigmentation of the SN. The weight of the brain was 1365g. The following sections were examined: mesencephalon, medulla oblongata, pons, vermis superior, dentate nucleus, frontal cortex, lentiform nucleus, gyrus cinguli, amygdala, hippocampus, thalamus, parietal lobe, occipital lobe (sulcus calcarinus), and striatum. The microscopic exam revealed neuron loss and gliosis in SN without LB. There were no signs of pathological changes in medulla oblongata or cerebellum. Slight small vessel changes were found in the cortex, amygdala, hippocampus, basal ganglia, and thalamus, without any neuronal loss or gliosis. Immunohistochemical staining for tau showed only a very few immunoreactive neurons and diffuse amyloid plaques and meningeal reaction compatible with cerebral amyloid angiopathy. Immunohistochemical stains for alpha-synuclein and ubiquitin did not show any LBs in the brain stem or cortex. Hippocampus showed light neurofibrillary degeneration but it was negative for tau and alpha-synuclein in the medulla oblongata including mesencephalon and nucleus lentiformis.

## 4. Discussion 

Here we present a patient with typical motor symptoms, an excellent response to levodopa, and he was successfully treated with DBS; thus his clinical diagnosis of PD was clear.* Parkin* mutation carriers have often young age at disease onset, benign long disease course, and long duration without dementia as it was seen in this case. The autopsy revealed no LB pathology consistent with most of the findings in* Parkin* mutation carriers [11–17, 23, 24]. Previous autopsy reports of patients with PD due to mutations in* Parkin* all had a young age of disease onset (Mean AAO= 25.4 years, range 18-34) with a benign disease course. Other case reports presented patients with diffuse LB pathology, one homozygous carrier had age at disease onset 61 years [22], another with 33 years [23], and the other five patients had compound heterozygous mutations [19–21, 24] with some later disease onset compared to patients without LB (Mean AAO: 46,3 years, range 33-61). Based on these findings it could be hypothesized whether homozygous mutations cause pure neuron loss without LBs while the compound heterozygous mutations might present with LB pathology. Because of very few cases it is difficult to conclude, genotype-phenotype correlations are challenging; the same mutation might present with various clinical phenotype as it has also been described in patients carriers* LRRK2 *p.G2019S. Even though the phenotype of* LRRK2* mutation carriers are similar to idiopathic PD cases, the neuropathology is heterogeneous and often without LBs [9]. 

A point mutation (p.R275W) in the* Parkin *gene has been described in four heterozygous cases with LBs [19, 24, 25] and has been observed to result in an unusual distribution of the protein, forming aggresomes, in cultured neurons [26]. It is unknown whether this mutation influence formation of LBs or if it is a random occurrence. Olanow et al. summarized a hypothesis about the formation of LBs similar to aggresomes [27]. Aggregated proteins in the cells induce the proteolytic system by forming aggresomes to degrade the toxic proteins. Overwhelming aggregation might keep on expending aggresomes leading to formation of LBs. Parkin is an ubiquitin ligase complex that mediates protein degradation in the cell. Hence,* Parkin* mutations might render the ubiquitin proteasome system dysfunctional leading to a lack of protective LB formation and hence, causing severe neuron loss. Genetic studies of PD have led to a vast insight into basal mechanisms, highlighted by the discovery of alpha-synuclein (*SNCA*), the main constituent of LBs. It is still not clear whether the formation of LBs leads to neurodegeneration or represents a neuroprotective process.

Several autopsy studies have reported incidental LB pathology in healthy elderly and it is still not clear whether they represent a preclinical stage or represents incidental findings. A Finnish brain bank study screened for alpha-synuclein inclusions, Braak stages 4 to 6. A total of 14% of cases were in this group. Almost all cases who had been diagnosed with Parkinson had LBs but only about 50% of the cases with incidental LBs were healthy without any movement disorders or cognitive deficits [28].

Autosomal recessive parkinsonism, most often caused by mutations in the* Parkin *gene, results in different clinical phenotype compared to idiopathic PD, with pure motor symptoms and a more benign disease course. The basal mechanisms are probably different as mitochondrial and proteasomal dysfunction might be more involved causing more isolated nigral affection without classical LB pathology. Because of these prominent difference from the idiopathic cases and often a positive family history which is an exclusion criteria in the UK Brain Bank PD diagnostic criteria it has been suggested to identify these cases as nigropathies as a separate entity from PD [29].

The movement disorder society has newly reviewed the diagnostic criteria of PD and describes two different certainty levels of the clinical diagnose: clinically established or clinically probable PD but no guidelines regarding the neuropathology [3]. According to this revision PD is a clinical syndrome and the diagnosis is based on the clinical signs to offer correct treatment. As in the presented case the patient had excellent levodopa response and later on received DBS because of severe dyskinesias with good effect. An earlier study showed that within the DBS group there was much higher prevalence of the genetic mutation carriers compared to a PD population without surgery, a probable reason being a more isolated motor subtype in these patients [30].

The introductory paper to this revision on the other hand has made some considerations regarding neuropathology [31], although the gold standard for diagnosis is not useful in the clinical setting as it is not applicable while a person is alive but pathological findings open up for basal pathomechanisms which might be a target for therapy. Ongoing studies evaluating effect of therapies developed for halting alpha-synuclein aggregation which probably might be effective in cases with LB pathology but not in pure neuron loss cases. Today we do not have sensitive biomarkers to detect ongoing neurodegenerative changes and a further selection of patients based on different progression rate and different clinical subtypes are also needed in order to be optimally treated.

## 5. Conclusion 

This case report confirms the importance of correlating clinical diagnostics to treatment in combination with neuropathological findings. Early onset* Parkin* cases have classical PD phenotype but do not develop dementia with Lewy bodies. Further studies are needed to establish good biomarkers for brain pathology and in patients with identical clinical features.
